# Comparison of *In Vitro* Endocrine Activity of Phthalates and Alternative Plasticizers

**DOI:** 10.1155/2021/8815202

**Published:** 2021-02-09

**Authors:** Hélène Moche, Aouatif Chentouf, Sergio Neves, Jean-Marc Corpart, Fabrice Nesslany

**Affiliations:** ^1^Institut Pasteur De Lille, 59019 Lille Cedex, France; ^2^ROQUETTE Company, 62136 Lestrem, France

## Abstract

Because of the deleterious effects of phthalates, regulations have been taken to decrease their use, and the needs for alternatives are increasing. Due to the concerns about the endocrine-disrupting properties of phthalates, it was deemed necessary to particularly investigate these effects for potential substitutes. In this study, we compared the *in vitro* endocrine activity of several already used potential alternative plasticizers (DEHT, DINCH, and TOTM) or new substitutes (POLYSORB® isosorbide and POLYSORB® ID 46) to one of 2 phthalates, DEHP and DINP. Effects of these chemicals on 3 common mechanisms of endocrine disruption, i.e., interaction with estrogen receptors (ER), androgen receptors (AR), or steroidogenesis, were studied using extensively used *in vitro* methods. In the E-Screen assay, only DEHP moderately induced MCF-7 cell proliferation; none of the other tested substances were estrogenic or antiestrogenic. No androgenic or antiandrogenic activity in MDA-kb2 cells was shown for any of the tested phthalates or alternatives. On the other hand, both DEHP and DINP, as well as DEHT, DINCH, and TOTM, disrupted steroidogenesis in the H295R assay, mainly by inducing an increase in estradiol synthesis; no such effect was observed for POLYSORB® isosorbide and POLYSORB® ID 46.

## 1. Introduction

Phthalates or phthalic acid esters are a family of synthetic organic chemicals that are mainly used as plasticizers in the polymer industry. They are notably used for the manufacture of polyvinylchloride (PVC), making it flexible and easily processable. Phthalates are thus used to produce a wide variety of articles such as building products, car products, clothing, sport equipment, luggage, toys, and medical devices [1–4].

Phthalates are not chemically bound to the PVC polymer, and thus, they can migrate from products upon contact with liquids or fats or under temperature or pH variations. They can thus be released into the surrounding environments [3, 5], and human exposure can occur through oral ingestion, inhalation, and dermal or parenteral routes [6, 7]. Some phthalates are described as having endocrine-disrupting properties: indeed, many studies showed that some phthalates have antiandrogenic activities and induce alterations in male reproductive-tract development after in utero or perinatal exposure in male rats [8–10]. A review [4] concluded that phthalate exposure at levels observed in human populations may have male reproductive effects, particularly regarding bis (2-ethylhexyl) phthalate (DEHP) and dibutyl phthalate (DBP).

Because of increasing concerns regarding their deleterious effects, regulations have been taken to decrease phthalate use. Four phthalates are now included in Annex XIV of the European Regulation on Registration, Evaluation, Authorisation and Restriction of Chemicals (REACH) listing the substances of very high concern requiring authorization before use and are classified as toxic for reproduction (category 1B): benzyl butyl phthalate (BBP), DEHP, DBP, and diisobutyl phthalate (DIBP). In Europe, some phthalates are restricted for use in toys and childcare articles in the REACH Regulation (Annex XVII): DEHP, DBP, and BBP. Following the recent publication of an amendment to Annex XVII [11], the same restrictions will apply to DIBP after July 2020, and these 4 phthalates will be restricted in plasticized materials in a wider array of products (not only toys and childcare articles). Thereby, DEHP, DBP, BBP, and DIBP are restricted in the insulation of electrical cables and wires in the RoHS (Restriction of Hazardous Substances) directive. Some restrictions on the use of phthalates in materials in contact with foodstuffs are defined by Directive 2007/19/EC: a ban on DIBP, specific migration limits for food for DEHP, DBP, BBP, and (together with other substances) DIDP and DINP. Moreover, diisononyl phthalate (DINP), diisodecyl phthalate (DIDP), and di-n-octyl phthalate (DNOP) are restricted in toys and childcare articles which can be placed in the mouth by children (Annex XVII to REACH). Similar restrictions regarding several phthalates are applicable in the USA [12]. Also, some phthalates (e.g., DBP, BBP, and DEHP) are prohibited in cosmetic products [13].

Due to the toxic effects and restriction of the use of various phthalates, there is a need for substitution. A number of alternative plasticizers with different chemical structures are either already used or in development. Among them, 1,2-cyclohexanedicarboxylic acid diisononyl ester (DINCH), tris-2-ethylhexyl trimellitate (TOTM), and bis-2-ethylhexyl terephthalate (DEHT) are already used as phthalate substitutes in a wide variety of applications [14]. Terephthalates are very similar to phthalates with two ring substitutions occupying para-positions instead of ortho-positions. DEHT, a structural isomer of DEHP, is the most common terephthalate and is used as a commercial alternative in a wide range of applications such as in plastic toys and childcare articles, films, pavement, stripping compounds, vinyl products, and beverage closures [14]. The trimellitate TOTM is used in high-temperature applications such as PVC cables with significantly improved extraction and migration resistance relative to other DEHP alternatives [14]. DINCH is used as an alternative plasticizer in high volumes and replaces phthalates such as DEHP and DINP in medical devices, toys, and food packaging materials.

As plant alternatives to phthalates, ROQUETTE Company has developed a 100% bio-sourced plasticizer, POLYSORB® ID 46. POLYSORB® ID 46 is a blend of diesters obtained from the esterification of isosorbide with plant-based fatty acids. POLYSORB® ID 46 is composed of the following constituents: (3R,3aR,6S,6aR)-hexahydrofuro [3,2-b]furan-3,6-diyl dioctanoate, (3S,3aR,6R,6aR)-6-(octanoyloxy)hexahydrofuro [3,2-b]furan-3-yl decanoate, (3R,3aR,6S,6aR)-6-(octanoyloxy)hexahydrofuro [3,2-b]furan-3-yl decanoate, and (3R,3aR,6S,6aR)-hexahydrofuro [3,2-b]furan-3,6-diyl bis(decanoate). This isosorbide derivative can be used to plasticize PVC in particular. POLYSORB® isosorbide is a high purity grade monomer specifically designed for the manufacture of many polymeric and nonpolymeric derivatives such as POLYSORB® ID 46. Used either directly as a very pure monomer or in the form of a derivative (functionalized monomer or plasticizer), POLYSORB® isosorbide offers valuable solutions to performance materials markets: polyesters, polycarbonates, PVC, composites and coatings, polyurethanes, TPU, etc. POLYSORB® ID 46 offers the performance of standard general-purpose primary plasticizers and outstanding compatibility and processability with PVC resins. Due to its great efficiency, it can be considered as an alternative of choice to standard petrochemical-based plasticizers. Registered in accordance with chemical control laws in main plastic producing areas, POLYSORB® ID 46, which has already been the subject of many toxicological studies (no effects were observed in the *in vitro* and *in vivo* genotoxicity studies, developmental toxicity study, and oral 90-d repeated dose toxicity study [15]), is recommended for applications in direct contact with end-users (hospital floorings, decorative indoor surfaces, school furniture, etc.).

Due to the concerns about the endocrine-disrupting properties of phthalates, it was deemed necessary to investigate these effects for POLYSORB® ID 46 and other potential phthalate substitutes. In this study, we aimed to compare the *in vitro* endocrine activity of several already used potential alternative plasticizers (DEHT, DINCH, and TOTM) or the new substitute POLYSORB® ID 46 to one of 2 phthalates, DEHP and DINP in a battery of *in vitro* assays. We also included in the study the monomer POLYSORB® isosorbide used in the manufacture of POLYSORB® ID 46. Effects of these chemicals on 3 common mechanisms of endocrine disruption, i.e., interaction with estrogen receptors (ER), androgen receptors (AR), or steroidogenesis, were studied using extensively used *in vitro* methods: the MCF-7 proliferation assay (E-Screen assay) [16], the AR transactivation assay using the MDA-kb2 cell line [17], and the H295R steroidogenesis assay (Organisation for Economic Co-operation and Development (OECD) Test Guideline No. 456 [18]. The MDA-kb2 assay is similar to the stably transfected human androgen receptor transcriptional activation assay for detection of androgenic agonist and antagonist activity of chemicals using the AR-EcoScreen™ cell line, described in the OECD Test Guideline No. 458 [19]. All these assays are included in level 2 of the OECD Conceptual Framework for Testing and Assessment of Endocrine Disrupting Chemicals [20] corresponding to “*in vitro* assays providing data about the selected endocrine mechanism(s)/pathway(s).”

## 2. Materials and Methods

### 2.1. Chemicals

Bis(2-ethylhexyl) phthalate (DEHP) was from Merck, diisononyl phthalate (DINP), bis(2-ethylhexyl) terephthalate (DEHT), and tris(2-ethylhexyl) trimellitate (TOTM) were from Sigma-Aldrich (Saint-Quentin Fallavier, France), and 1,2-cyclohexanedicarboxylic acid diisononyl ester (DINCH) was from BASF. All these compounds had a purity of at least 96%. POLYSORB® isosorbide (purity >99.5%) and POLYSORB® ID 46 (purity >96% (batch YE008) or 94.5% (batch YE34)) were synthesized by ROQUETTE Company. Dulbecco's modified Eagle medium (DMEM) with and without phenol red, fetal bovine serum (FBS), Leibovitz's L-15 medium with and without phenol red, 1 : 1 mixture of Dulbecco's Modified Eagle's Medium and Ham's F-12 Nutrient (DMEM/F12) culture medium without phenol red, phosphate-buffered saline (PBS), and charcoal-dextran stripped FBS (CD/FBS) for E-Screen assay were from Gibco (Life Technologies, Paisley, UK). 17*β*-estradiol, ICI 182,780 (fulvestrant), dihydrotestosterone (DHT), hydroxyflutamide, forskolin, prochloraz, and CD/FBS for MDA-kb2 assay were from Sigma-Aldrich (Saint-Quentin Fallavier, France).

Phthalates and substitutes were solubilized in DMSO or ethanol. When diluted in cell culture media for experiments, solvent concentration never exceeded 0.1%.

### 2.2. E-Screen Assay

The MCF-7 cell proliferation assay (E-Screen assay) was performed according to the method described by Soto et al. [16] with modifications [21].

#### 2.2.1. Cell Culture

Estrogen-responsive MCF-7 cells (BUS stock) were obtained from Pr. Ana Soto (Tufts University, Boston, USA). MCF-7 cells were grown and maintained in DMEM culture medium supplemented with 5% FBS at 37°C with 5% CO_2_ and 95% humidity.

#### 2.2.2. E-Screen Assays

Experiments were performed in 24-well plates (Falcon, Corning) in which cells were seeded in a culture medium at a density of 15000 cells/well and allowed to attach for 24 h. At the end of the attachment period, plated cells were inspected with a microscope to ensure that they were in good condition (attachment, morphology) prior to dosing. Cells were washed with PBS and exposed to dosing solutions in an experimental medium (DMEM without phenol red supplemented with 5% CD/FBS) and incubated for 120 hours at 37°C with 5% CO_2_ and 95% humidity. For each compound, two independent agonist and antagonist assays were performed as described below, with a potentially adjusted range of concentrations in the 2^nd^ assay.

*(1) Agonist Assays*. Each test plate included 7 concentrations of phthalate or substitute and solvent control (SC: DMSO or ethanol 0.1%) in triplicate. Each test run also included a positive control plate, in which cells were exposed to 5 concentrations of the reference estrogen 17*β*-estradiol (10^−9^–10^−10^–10^−11^–10^−12^–10^−13^ M) in triplicate wells.

*(2) Antagonist Assays*. Dosing solutions were prepared in an experimental medium supplemented with a fixed concentration of E2 (1 pM). Each test plate included 5 concentrations of phthalate or substitute, a solvent control (SC: DMSO or ethanol 0.1%), and ICI 182,780 (1 nM) as reference antiestrogenic compound, each in triplicate. Each test plate also included 3 replicate wells with the solvent diluted in an experimental medium without E2.

*(3) Competitive Confirmation Agonist Assay*. To investigate ER involvement in DEHP-induced MCF-7 cell proliferation, the effect of pure antiestrogen ICI 182,780 on cell proliferation induced by DEHP was examined [21]. ICI 182,780 (final concentration 1 nM) was added to the medium in the presence of DEHP at the concentration inducing the highest cell proliferation. Moreover, E2 (10 pM) was added as a positive control.

*(4) Competitive Confirmation Antagonist Assay*. To investigate ER involvement in DINP- and DEHT-induced decrease in E2-induced MCF-7 cell proliferation, the effect of increasing concentrations of E2 on the inhibition of cell proliferation by DINP and DEHT was examined. Cells were exposed to 10, 100, and 1000 pM E2 in an experimental medium supplemented with a fixed concentration of DINP (30 *μ*M) or DEHT (300 *μ*M), these concentrations inducing the maximal decrease in E2-induced cell proliferation. As means of comparison, the solvent, the pure antiestrogen ICI 182,780 (1 nM), and CCCP (carbonyl cyanide 3-chlorophenylhydrazone, 2.5 *μ*M) as cytotoxic reference were also used.

#### 2.2.3. Assessment of Cell Proliferation

For all types of assays, after the 5-day exposure period, determination of cell density in each well was performed by measuring total protein contents using the sulforhodamine B (SRB) assay. The dosing medium was discarded, and cells were fixed with cold 10% (w/v) trichloroacetic acid (Sigma-Aldrich) for 30 min at 4°C. Wells were then washed 4 times with demineralized water and air-dried. Staining was performed with 0.4% (w/v) solution of SRB (Sigma-Aldrich) in 1% (v/v) acetic acid for 10 min. Wells were washed with 1% acetic acid to remove unbound SRB and air-dried. Bound SRB dye was solubilized in Tris base solution (10 mM, pH 10.5), and plates were agitated on a plate shaker until complete dissolution of the dye. Optical density (OD) was determined using SPECTROstar® Nano equipment (BMG Labtech, Champigny s/Marne, France) at 530 nm with a 690 nm reference wavelength. To take into account the proteins contained in the experimental medium, the average OD530-690 value of 3 background wells incubated with the experimental medium without cells was subtracted from the OD530-690 value of each well.

The proliferative effect, corresponding to the ratio of the OD530-690 value obtained for each concentration of the compound and the OD530-690 value in solvent control, was calculated for each compound concentration. Data for each phthalate or substitute concentration were expressed as mean ± standard deviation (SD) for triplicate wells.

#### 2.2.4. Statistical Analysis

In agonist and antagonist assays, statistical analyses were performed using Dunnett's test comparing the cell proliferation induced by each concentration of the test item with the cell proliferation observed in solvent control for agonist assays, and solvent control with E2 for antagonist assays. In the confirmation agonist assay, differences between proliferative effects observed with and without ICI 182,780 were compared using the Mann–Whitney test. The statistical software GraphPad InStat version 3.10 was used for the analyses. Differences were considered significant at *p* ≤ 0.05.

### 2.3. MDA-kb2 Androgen Receptor Transactivation Assay

The AR transactivation assay using MDA-kb2 cells originally described by Wilson et al. [17] was performed essentially according to the protocol modified by Ermler et al. [22]. The method described in the OECD TG No 458 [19] was used for results analysis and interpretation.

#### 2.3.1. Cell Culture

The MDA-kb2 cell line (ATCC CRL-2713) was obtained from the American Type Culture Collection (ATCC, Manassas, VA, USA). MDA-kb2 cells were grown and maintained in Leibovitz's L-15 medium supplemented with 10% fetal bovine serum, 100 U/mL penicillin, and 100 *μ*g/mL streptomycin at 37°C without CO_2_.

#### 2.3.2. MDA-kb2 Assays

Before plating, cells were acclimated for 24 h in an experimental medium, composed of Leibovitz's L-15 medium without phenol red supplemented with 10% charcoal-dextran-treated fetal bovine serum and penicillin/streptomycin. Cells were then plated in 96-well white luminometer plates at a density of 5 × 10^4^ cells/well in 50 *μ*L experimental medium and allowed to attach for 24 h. Cells were also identically plated in standard 96-well plates dedicated to the MTT cytotoxicity assay. At the end of the attachment period, 2x-concentrated dosing solutions were directly added in triplicate to the wells of both white and standard plates. Plates were briefly agitated on a plate shaker and were then returned to the incubator for 20 to 24 h. For each compound, two independent agonist and antagonist assays were performed as described below, with a potentially adjusted range of concentrations in the 2^nd^ assay.

*(1) Agonist Assays*. Dosing solutions were prepared in an experimental medium and contained solvent, a test compound, or the reference androgen DHT at twice the intended final concentrations. Seven dilutions of each phthalate or substitute were prepared, as well as 7 dilutions of DHT (final concentrations from 10^−12^ to 10^−6^ M).

*(2) Antagonist Assays*. Dosing solutions were prepared in an experimental medium supplemented with 0.5 nM DHT (twice the intended final concentration of 0.25 nM) and contained solvent, a test compound, or the reference antiandrogen hydroxyflutamide at twice the intended final concentrations. Six dilutions of each phthalate or substitute were prepared, as well as 6 dilutions of hydroxyflutamide (final concentrations from 10^−10^ to 10^−5^ M). Moreover, a solvent control without DHT was added in 3 wells.

*(3) Competitive Confirmation Antagonist Assay*. In case discrimination between actual antagonist effect and nonspecific effect (e.g., cytotoxicity-related effect) was considered necessary, a competitive confirmation antagonist assay was performed with the test item concentration inducing the maximum effect in standard antagonist assays and increasing concentrations of DHT. Dosing solutions were prepared in an experimental medium supplemented with the test item at twice the intended concentration and contained increasing concentrations of the reference androgen DHT (final concentrations 0–0.1–0.25–0.5–1–5 and 10 nM). Moreover, dosing solutions containing the increasing concentrations of DHT were also prepared in an experimental medium supplemented with solvent or with hydroxyflutamide (final concentration 1 *μ*M) or CCCP (carbonyl cyanide 3-chlorophenylhydrazone, final concentration 10 *μ*M) as antiandrogenic and cytotoxic reference compounds.

#### 2.3.3. Assessment of Cell Viability

At the end of the 20 to 24 h exposure period, cell viability was measured in dedicated plates using the colorimetric MTT (3-(4,5-dimethylthiazol-2-yl)-2,5-diphenyltetrazolium bromide) assay [23]. All wells were emptied, and a solution of MTT (0.5 mg/mL) in medium without phenol red was added to each well. Plates were incubated for 2–2.5 hours at 37°C. The medium containing MTT was then removed, and a mix of isopropanol/HCl 1 N was added to the wells. The plates were placed under agitation on a shaker plate until the complete dissolution of formazan crystals. Optical density was determined using SPECTROstar equipment at 570 nm with a 640 nm reference wavelength.

Cell viability was expressed relative to the average response in the solvent control wells, and only concentrations inducing at least 80% viability were analyzed.

#### 2.3.4. Measurement of Luciferase Activity

After the 20–24 h exposure period, luciferase activity was measured in the white 96-well plates. Plates were emptied, and DMEM without phenol red and Steady-Glo® Luciferase Assay System reagent (Promega, Madison, USA) were added to the wells. Plates were agitated for 10 min at 500 rpm on an orbital plate shaker, and luminescence was measured using a luminometer (Tecan Infinite® Lumi, Tecan, Switzerland). Luminescence signals were corrected for background (wells without cells) and expressed in Relative Light Units (RLU).

#### 2.3.5. Analysis of Results

Results' analysis and interpretation were performed according to the method described in OECD TG 458 [19]. Relative transcriptional activity (RTA) was calculated for each concentration of the phthalates and substitutes. In agonist assays, RTA was relative to DHT 10 nM, set to 100%, and calculated by the following equation:

(1) $$\begin{array}{c} \text{RTA}\left(\%\right)=\frac{\left(\text{RLU in the well}-\text{average RLU in SC wells}\right)}{\left(\text{average RLU in }10\text{ nM DHT wells}-\text{average RLU in SC wells}\right)}\times 100. \end{array}$$

In antagonist assays, RTA was relative to solvent control with DHT, set to 100%, and was calculated by the following equation:

(2) $$\begin{array}{c} \text{RTA}\left(\%\right)=\frac{\left(\text{RLU in the well}-\text{average RLU in SC wells}\right)}{\left(\text{average RLU in SC}+\text{DHT wells}-\text{average RLU in SC wells}\right)}\times 100. \end{array}$$

For each concentration, RTA was expressed as mean ± SD of triplicate wells. If appropriate, the concentrations inducing an RTA corresponding to 10% and 50% of DHT 10 nM effect (PC_10_ and PC_50_) were determined in the agonist assays, and the concentrations inducing 30% and 50% inhibition of transcriptional activity induced by solvent control with DHT (IC_30_ and IC_50_) were determined in the antagonist assays. A substance is considered positive in the agonist assay if the PC_10_ can be calculated in at least 2 of 2 or 3 assays; a substance is considered positive in the antagonist assay if the IC_30_ can be calculated in at least 2 of 2 or 3 assays.

In the competitive confirmation antagonist assays, relative luciferase activity compared to the solvent control (set to 1) is calculated by dividing the RLU measured in each well by the average RLU measured in SC wells.

### 2.4. H295R Steroidogenesis Assay

H295R steroidogenesis assay was performed according to the OECD TG No. 456 [18] and Hecker et al. [24, 25]. Two independent assays were performed, with a potentially adjusted range of concentrations in the 2^nd^ assay.

#### 2.4.1. Cell Culture

The H295R human adenocarcinoma cell line (NCI-H295R, ATCC CRL-2128) was purchased from the ATCC (Manassas, VA, USA). Cells were cultured in a 1 : 1 mixture of Dulbecco's Modified Eagle's Medium and Ham's F-12 Nutrient (DMEM/F12) without phenol red, supplemented with 2.5% Nu-Serum™ (Corning) and 1% ITS + premix (insulin, transferrin, selenous acid, bovine serum albumin, and linoleic acid, Corning) at 37°C with 5% CO_2_ and 95% humidity. After thawing, H295R cells were grown for 5 to 10 passages before starting the experiments.

#### 2.4.2. Exposure

Experiments were performed in 24-well plates in which cells were initially seeded at a density of 250000 cells/well and allowed to attach for 24 h. Test plates included 7 chemical concentrations and solvent control (SC: DMSO or ethanol 0.1%) in triplicate. Quality control (CQ) plates were included in each test run and comprised 2 concentrations of the steroidogenesis inducer forskolin (1 and 10 *μ*M), 2 concentrations of the inhibitor prochloraz (0.1 and 1 *μ*M), a blank (culture medium only)n and an SC (DMSO 0.1%) in triplicate, as well as 6 wells dedicated to cytotoxicity reference, in which ethanol 70% was added at the end of the exposure. Test and QC plates were incubated at 37°C with 5% CO_2_ for 48 h. At the end of the 48 h exposure period, the medium in each well was collected and centrifuged to eliminate particles before hormone measurement (3000 rpm, 10 min). To prevent cells from drying, PBS was added to each well before cell viability assessment.

#### 2.4.3. Cell Viability Assessment

Cell viability in each well was analyzed using the MTT assay as described previously. Cell viability was expressed relative to the average response in the SC wells, which is considered 100% viable cells, and was calculated using the following formula, with EtOH wells being considered 100% dead cells:

(3) $$\begin{array}{c} \text{Relative survival}\left(\text{\%}\right)=\frac{\left(\text{OD in well}-\text{average OD in EtOH wells}\right)}{\left(\text{average OD in SC wells}-\text{average OD in EtOH wells}\right)}\times 100. \end{array}$$

Wells with viability lower than 80% relative to the average viability in the solvent controls were not included in the final data analysis. For wells with viability between 80% and 85% relative to the average viability in the solvent controls, hormone concentrations were corrected according to the corresponding viability in order to avoid any misinterpretation of potential hormonal modulations.

#### 2.4.4. Hormone Measurements

Testosterone (T) and estradiol (E2) measurements were performed on the centrifuged supernatants using appropriate ELISA kits (Testosterone and Estradiol Parameter™ ELISA kits, R&D Systems) according to the manufacturer's instructions, with a few modifications needed for optimization: T and E2 standards and samples for T analysis were diluted in cell culture medium without serum instead of diluents provided in the kits. Prior to the beginning of the assay, interference tests were performed to test for potential interference of the test item with the hormone measurement systems.

To evaluate the relative change in hormone production for each phthalate or substitute concentrations, results are expressed as fold changes relative to the mean solvent control in each test plate. For each hormone, fold changes were calculated as follows for each well:

(4) $$\begin{array}{c} \text{Fold Change}=\frac{\left(\text{Hormone concentration in each well}\right)}{\left(\text{Mean hormone concentration in solvent control wells}\right)}. \end{array}$$

Data for each phthalate or substitute concentration were expressed as mean ± standard deviation (SD) of fold changes for triplicate wells.

#### 2.4.5. Statistical Analysis and Result Interpretation

The analysis of the results was performed according to the method described in OECD TG 456 [18]. Prior to conducting statistical analyses, the assumption of normality was evaluated using the Kolmogorov–Smirnov test. When data were normally distributed, differences between chemical concentration groups and solvent controls were analyzed using Dunnett's test. When data were not normally distributed, differences between chemical concentration groups and solvent controls were analyzed using the Mann–Whitney test. The statistical software GraphPad InStat version 3.10 (GraphPad Software, San Diego California, USA) was used for the analyses. Differences were considered significant at *p* ≤ 0.05.

A substance is considered positive if the fold change for E2 and/or T concentration is statistically different from the solvent control at 2 adjacent concentrations in at least 2 independent assays [18].

## 3. Results

### 3.1. Estrogenic Activity of Phthalates and Substitutes in the E-Screen Assay

**Figure 1:**
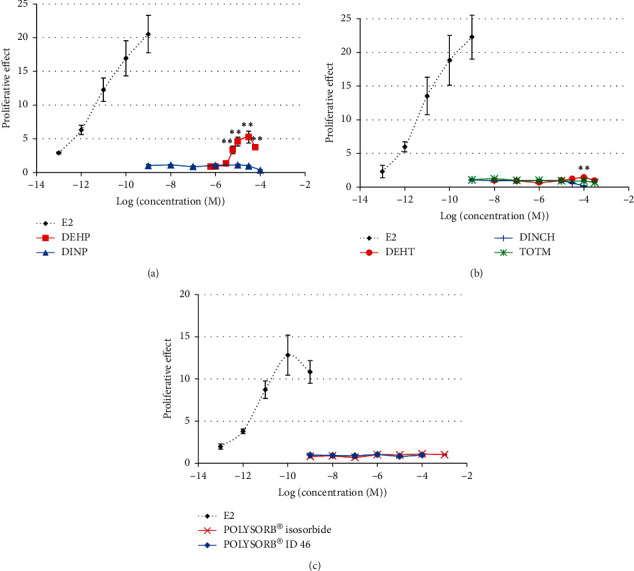
Estrogenic activity of phthalates and substitutes in the E-Screen agonist assay. MCF-7 cells were exposed for 120 h to different concentrations of DEHP, DINP (a), DEHT, DINCH, TOTM (b), POLYSORB® isosorbide, or POLYSORB® ID 46 (c). Results represent the mean proliferative effect compared to SC (=1) ±SD of 3 replicates from one representative assay out of 2 assays. *∗∗*Statistically significant increase compared to SC (*p* < 0.01). In the competitive confirmation assay (Figure 2), the cell proliferation induced by 30 *μ*M DEHP was statistically significantly decreased in the presence of the reference antiestrogen ICI 182,780. This confirms that the DEHP-induced MCF-7 cell proliferation was mediated by an estrogenic mechanism.

DEHP was the only compound inducing a significant increase in MCF-7 cell proliferation compared to the solvent control in the agonist E-Screen assay (Figure 1(a)). This statistically significant increase was, however, moderate, with a maximum proliferative effect of 5.25 at the DEHP concentration of 3.10^−5^ M, corresponding to about 30% of the proliferative effect induced by estradiol. A statistically significant increase in cell proliferation was also observed with DEHT (Figure 1(b)), but the proliferative effect was very weak (lower than twice the proliferation in the solvent control and lower than 10% of the proliferative effect induced by E2), and this effect, observed at the concentration of 10^−4^ M only, was not considered relevant. None of the other substitutes, or DINP, induced any significant increase in MCF-7 cell proliferation at concentrations ranging from 10^−9^ M up to 10^−4^ to 10^−3^ M (Figure 1).

### 3.2. Antiestrogenic Activity of Phthalates and Substitutes in the E-Screen Assay

**Figure 3:**
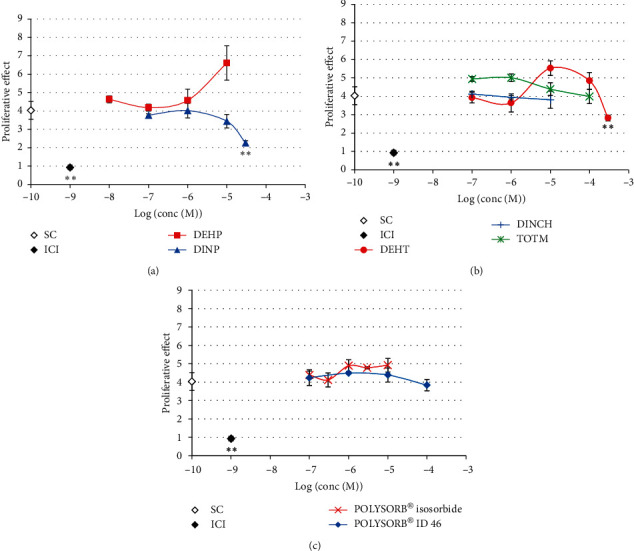
Antiestrogenic activity of phthalates and substitutes in the E-Screen antagonist assay. MCF-7 cells were exposed for 120 h to different concentrations of DEHP, DINP (a), DEHT, DINCH, TOTM (b), POLYSORB® isosorbide, or POLYSORB® ID 46 (c) in presence of 1 pM E2. Solvent (SC) and ICI 182,780 (ICI) were used as controls. Results represent the mean proliferative effect compared to the solvent control without E2 (=1) ±SD of 3 replicates from one representative assay out of 2 assays. ^*∗∗*^Statistically significant decrease compared to SC with E2 (*p* < 0.01).

**Figure 4:**
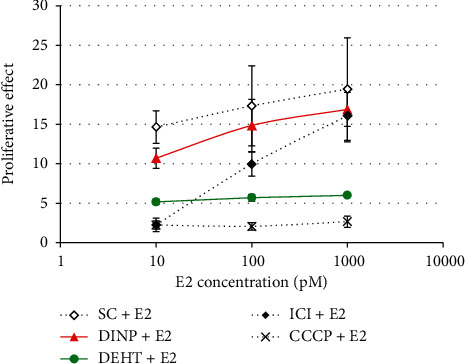
E-Screen antagonist competitive confirmation assay for DINP and DEHT. MCF-7 cells were exposed for 120 h to DEHT 300 *μ*M or DINP 30 *μ*M in presence of increasing concentrations of E2. ICI 182,780 (ICI) and carbonyl cyanide 3-chlorophenylhydrazone (CCCP) were used as antiestrogenic and cytotoxic reference compounds. Results represent the mean ± SD of 3 replicates.

No significant decrease in E2-induced MCF-7 cell proliferation was observed for DEHP, DINCH, TOTM, POLYSORB® isosorbide, or POLYSORB® ID 46 at concentrations ranging from 10^−8^ to 10^−7^ M up to 10^−5^ to 10^−4^ M. Only DINP and DEHT induced a statistically significant decrease in E2-induced MCF-7 cell proliferation (Figure 3). However, for both compounds, this was observed only at the highest concentration, i.e., DINP 3.10^−5^ M and DEHT 3.10^−4^ M, and in the competitive assay in presence of increasing concentrations of E2 (Figure 4), the effect was considered nonspecific. Indeed, the inhibition of proliferation observed for each E2 concentration between the solvent and DINP or DEHT did not decrease with increasing E2 concentrations, as was the case for ICI 182,780. Moreover, the inhibition induced by DINP 30 *μ*M was rather low.

### 3.3. (Anti)Androgenic Activity of Phthalates and Substitutes in the AR Gene Reporter Assay in MDA-kb2 Cells

**Figure 5:**
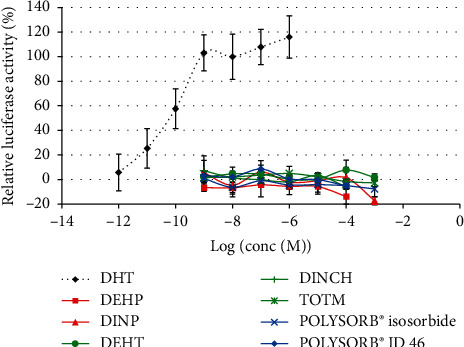
Androgenic activity of phthalates and substitutes in MDA-kb2 cells. MDA-kb2 cells were exposed for 20–24 h to different concentrations of DEHP, DINP, DEHT, DINCH, TOTM, POLYSORB® isosorbide, or POLYSORB® ID 46. Results represent the mean relative luciferase activity compared to DHT 10–8 M (=100%) ±SD of 3 replicates from one representative assay out of 2 assays.

Only concentrations inducing more than 80% viability compared to the control in the MTT assay were analyzed for AR activity. None of the tested compounds induced any *in vitro* AR agonist activity in the MDA-kb2 assay at concentrations ranging from 10^−9^ M to 10^−4^ or 10^−3^ M (Figure 5).

**Figure 6:**
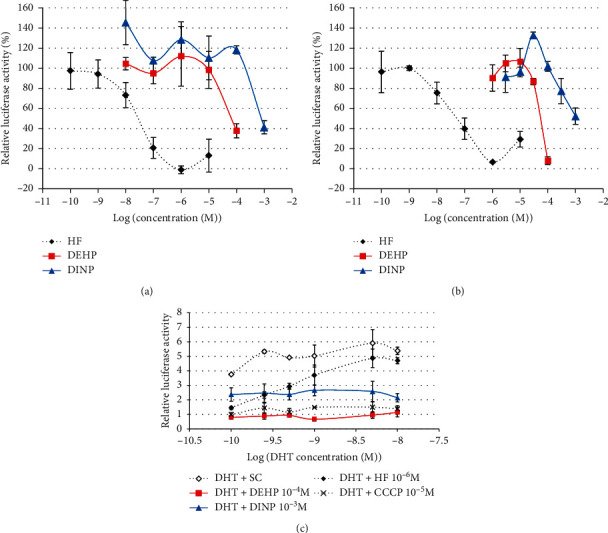
Antiandrogenic activity of phthalates DEHP and DINP in MDA-kb2 cells. (a, b) MDA-kb2 cells were exposed for 20–24 h to different concentrations of DEHP or DINP together with 0.25 nM DHT in 2 independent assays. Results represent the mean relative luciferase activity compared to the solvent control with 0.25 nM DHT (=100%) ±SD of 3 replicates in the 1st (a) or 2nd assay (b). (c) Competitive assay with increasing DHT concentrations. MDA-kb2 cells were exposed for 20–24 h to a single concentration of DEHP (10–4 M) or DINP (10–3 M) concomitantly with increasing concentrations of DHT. Solvent (SC), reference antiandrogen hydroxyflutamide (HF), and reference cytotoxic carbonyl cyanide 3-chlorophenylhydrazone (CCCP) were used as controls. Results represent the mean ± SD of 3 replicates.

**Figure 7:**
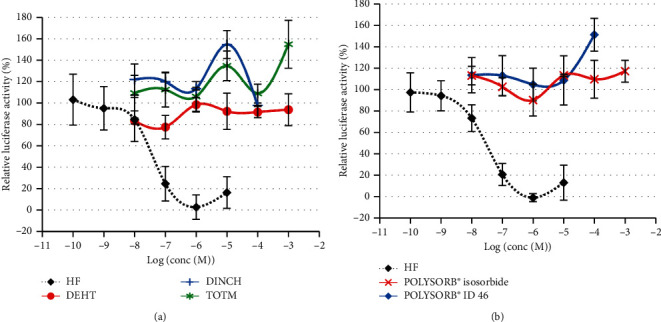
Antiandrogenic activity of phthalate substitutes in MDA-kb2 cells. MDA-kb2 cells were exposed for 20–24 h to different concentrations of (a) DEHT, DINCH, or TOTM or (b) POLYSORB® isosorbide or POLYSORB® ID 46 or the reference antiandrogen hydroxyflutamide (HF) together with 0.25 nM DHT. Results represent the mean relative luciferase activity compared to the solvent control with 0.25 nM DHT (=100%) ±SD of 3 replicates from one representative assay out of 2 assays.

A significant decrease in DHT-induced luciferase activity was observed only at the highest analyzed concentrations of both phthalates DEHP and DINP, i.e., 10^−4^ M and 10^−3^ M, respectively (Figures 6(a) and 6(b)). These decreases were not reversed by increasing concentrations of DHT (Figure 6(c)) and were thus not considered to be mediated by specific antiandrogenic mechanisms. None of the 5 studied substitutes induced any decrease in DHT-induced luciferase activity at concentrations ranging from 10^−8^ M to 10^−4^ or 10^−3^ M (Figure 7).

### 3.4. Effects of Phthalates and Substitutes on Estradiol and Testosterone Production in the H295R Steroidogenesis Assay

**Figure 8:**
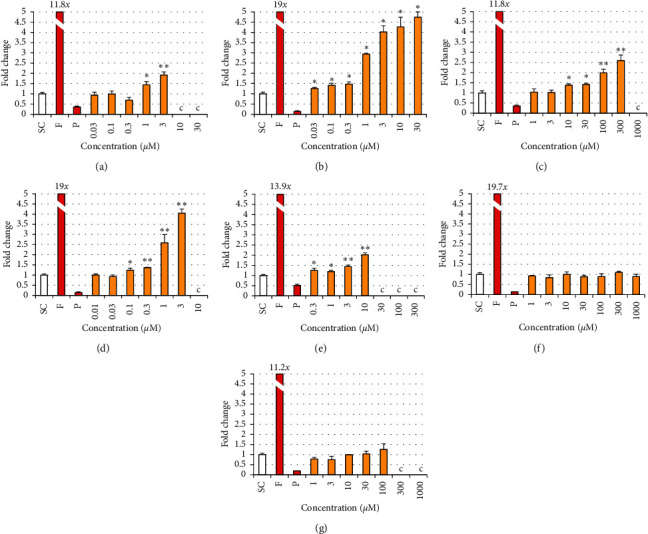
Estradiol production in H295R cells exposed to phthalates and substitutes. H295R cells were exposed for 48 h to different concentrations of each phthalate and substitute, solvent control (SC), and forskolin 10 *μ*M (F) and prochloraz 1 *μ*M (P) as reference inducer and inhibitor of steroidogenesis. (c) Cytotoxic concentration (viability < 80%) is not analyzed. Results represent the mean fold change in hormone concentration compared to SC (=1) ±SD of 3 replicates from one representative assay out of 2 assays. ^∗^*p* < 0.05, ^*∗∗*^*p* < 0.01. (a) DEHP. (b) DINP. (c) DEHT. (d) DINCH. (e) TOTM. (f) POLYSORB® isosorbide. (g) POLYSORB® ID 46.

**Figure 9:**
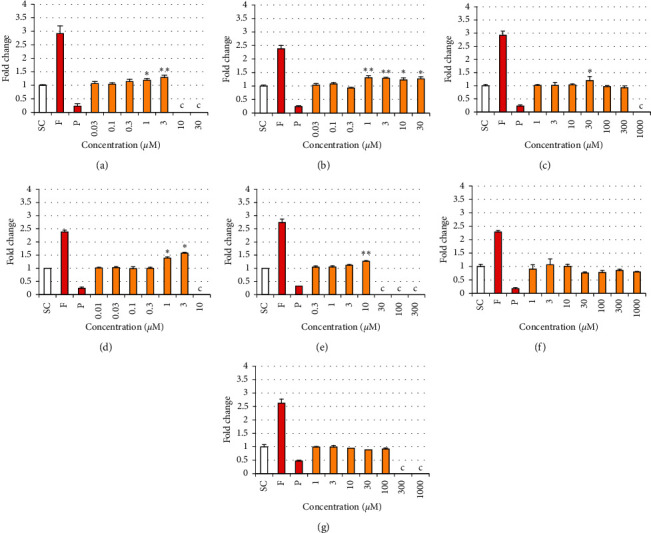
Testosterone production in H295R cells exposed to phthalates and substitutes. H295R cells were exposed for 48 h to different concentrations of each phthalate and substitute, solvent control (SC), and forskolin 10 *μ*M (F) and prochloraz 1 *μ*M (P) as reference inducer and inhibitor of steroidogenesis. Results represent the mean fold change in hormone concentration compared to SC (=1) ±SD of 3 replicates from one representative assay out of at least 2 assays. c: cytotoxic concentration (viability < 80%) not analyzed. ^*∗*^*p* < 0.05, ^*∗∗*^*p* < 0.01. (a) DEHP. (b) DINP. (c) DEHT. (d) DINCH. (e) TOTM. (f) POLYSORB® isosorbide. (g) POLYSORB® ID 46.

Only noncytotoxic concentrations were included in the analysis; concentrations inducing less than 80% viability compared to the control in the MTT assay were not analyzed for hormone production. DEHP induced a statistically significant increase in E2 production in H295R cells compared to solvent control at 2 adjacent concentrations, i.e., 1 and 3 *μ*M, the maximal effect (2-fold) being observed at 3 *μ*M (Figure 8). A weaker (maximum of 1.3-fold) but statistically significant increase in T production was also observed at the same concentrations (Figure 9). DINP also induced a statistically significant increase in E2 production at 7 adjacent concentrations, from 0.03 to 30 *μ*M, the maximal effect (4.7-fold) being observed at 30 *μ*M (Figure 8). A weak (1.3-fold) but statistically significant increase in T production was also observed from 1 to 30 *μ*M without any concentration-effect relationship (Figure 9).

DEHT induced a statistically significant increase in E2 production at 4 adjacent concentrations, from 10 to 300 *μ*M, the maximal effect (2.6-fold) being observed at the concentration of 300 *μ*M (Figure 8). The weak (1.2-fold) and sporadic effect observed in T production at the intermediate concentration of 30 *μ*M was not considered significant (Figure 9). DINCH induced a statistically significant increase in E2 production at 4 adjacent concentrations, from 0.1 to 3 *μ*M, the maximal effect (4-fold) being observed at the concentration of 3 *μ*M (Figure 8). Weaker but statistically significant increases in T production were also observed at 1 and 3 *μ*M (Figure 9), with a maximal effect of 1.6-fold induced at the concentration of 3 *μ*M. TOTM induced a statistically significant increase in E2 production at 4 adjacent concentrations, from 0.3 to 10 *μ*M, the maximal effect (2-fold) being observed at 10 *μ*M (Figure 8). The weak (1.3-fold) but statistically significant change in T production observed at only one single concentration (10 *μ*M) was not considered as significant (Figure 9).

POLYSORB® isosorbide and POLYSORB® ID 46 did not induce any significant change in either E2 or T productions at concentrations up to 1000 *μ*M and 100 *μ*M, respectively (Figures 8 and 9).

## 4. Discussion

Due to the restriction of the use of several phthalates, the production and use of substitutes are increasing. In the present study, we aimed at comparing the effects of 2 phthalates, DEHP and DINP, with phthalate substitutes, in *in vitro* assays allowing detecting (anti)estrogenic, (anti)androgenic, or steroidogenesis-disrupting compounds. The well-described E-Screen assay was used to detect (anti)estrogenic activity [16, 21]. To investigate (anti)androgenic activity, an AR transactivation assay using MDA-kb2 cells [17], similar to the assay described in the OECD TG 458 [19], was performed. Effects on steroidogenesis were studied with the H295R steroidogenesis assay described in the OECD TG 456 [18].

As phthalate substitutes, we studied DEHT, TOTM, DINCH, POLYSORB® isosorbide, and POLYSORB® ID 46.

Globally, our results for the phthalates DEHP and DINP showed that they both increased estradiol production in H295R cells; although statistically significant, the weak increases in testosterone production were considered of low relevance. Only DEHP was moderately estrogenic in the E-Screen assay, and neither of these 2 phthalates was androgenic or antiandrogenic in the MDA-kb2 assay. Our results for DEHP on steroidogenesis only partially correspond to the ones obtained by Mankidy et al. [26], who showed in H295R cells not only increased production of E2 but also decreased testosterone production. A decrease in testosterone production was also observed in another study in H295R cells and human testis explants [27]; on the opposite, Hadrup et al. [28] did not show any change in testosterone production in H295R cells. According to one of the postulated modes of action for DEHP, a significant decrease in testosterone production was expected in the steroidogenesis assay, which was not the case in our results. It should be noted that *in vivo*, DEHP is hydrolyzed to several metabolites. Among them, MEHP and 2-ethyl-hexanal have been shown to affect steroidogenesis in MA-10 mouse Leydig tumor cells [29]. The lack of a metabolic system in the *in vitro* assays for endocrine disruption is frequently pointed out, and it can be supposed that this could explain our results. However, Desdoits-Lethimonier et al. [27] showed that in human testis explants and H295R cells, DEHP was processed into MEHP, and that the latter was further processed into 5OH-MEHP.

Regarding our results in the E-Screen assay, the proliferative effect of DEHP in MCF-7 cells was also observed by Okubo et al. [30] and Tanay Das et al. [31], this latter study also reporting a proliferation of ER*α*-negative MDA-MB-231 cells, questioning the ER-dependency of the response. Our results rather suggest the opposite, as the MCF-7 cell proliferation induced by DEHP was inhibited in presence of the antiestrogen ICI 182,780. In the literature, results of ER gene reporter assays for DEHP are also heterogeneous: DEHP did not induce any estrogenic response in the MVLN transactivation reporter assay [26, 32] or ER*α* activation in hER*α*-HeLa9903 cells [33], ER*α*-HEK cells [34], or CV-1 cells [35]; DEHP was negative in a recombinant human ER*α* assay [33]. On the opposite, an agonist ER*α* activity was shown in a transactivation assay in CHO-K1 cells [36]. No antagonist activity was shown in ER*α* transactivation assays [34, 36] but DEHP exhibited ER*β*-mediated antiestrogenic activity [36].

Similarly, our results in the MDA-kb2 assay correspond to some of the results found in other studies. In MDA-kb2 cells, Engel et al. [34] did not show any agonist activity for DEHP, but it acted as an antagonist. Shen et al. [35] reported both androgenic and antiandrogenic activities for high concentrations of DEHP (10^−4^ M) in MDA-kb2 cells. In similar *in vitro* models, DEHP was neither androgenic nor antiandrogenic [36–39].

Less data was found in the literature regarding the other phthalate DINP. No data was found regarding *in vitro* steroidogenesis, but *in vivo*, Borch et al. [9] showed a decreased testosterone production in testes. To the best of our knowledge, DINCH has not been previously studied in E-Screen assays, but the results from other *in vitro* estrogenicity assays are globally in agreement with our findings in the E-Screen assay. Indeed, in ER*α* transactivation assays, DINP did not act as an agonist [32, 34, 36]; ER*α* antagonist activity was shown in some studies [34] but not in others [32, 36]. In *in vitro* AR transactivation assays in MDA-kb2 cells or others, no significant agonist or antagonist activities were observed for DINP [34, 36, 37]. This confirmed our results in MDA-kb2 cells.

Regarding the phthalate substitute DINCH, Engel et al. [40] reported in the H295R steroidogenesis assay slight and not statistically significant stimulations of estradiol synthesis at concentrations of 10 *μ*M and 100 *μ*M and of testosterone synthesis at the concentration of 100 *μ*M. In our results, however, DINCH induced a statistically significant increase in estradiol production in H295R cells. In another *in vitro* steroidogenesis model, DINCH had a biphasic effect on fetal Leydig cell testosterone production *in vitro*, increasing testosterone production at 10^−6^ M, and decreasing it at the high concentration of 10^−4^ M [41]. Similarly to our results in E-Screen assay and AR transactivation assay in MDA-kb2 cells, DINCH did not induce or inhibit ER*α*, ER*β*, or AR activity gene reporter assays performed in ER*α*-HEK, ER*β*-HEK, and MDA-kb2 cells [40].

Regarding the 2 other alternative plasticizers DEHT and TOTM, neither of them exhibited any estrogenic or antiestrogenic activity in the E-Screen assay or any androgenic or antiandrogenic activity in the AR gene reporter assay in MDA-kb2 cells. However, in the H295R steroidogenesis assay, they both induced a clear increase in cellular estradiol production.

Overall, when the comparison was possible, our results obtained in a battery of tests were similar to the published data.

We also included in this study a new substitute for phthalates, POLYSORB® ID 46, and the monomer used for its manufacture, POLYSORB® isosorbide. Similar to the other tested substitutes, neither of them was estrogenic or antiestrogenic in the E-Screen assay or androgenic or antiandrogenic in the AR gene reporter assay in MDA-kb2 cells. Interestingly, on the contrary to all other tested compounds (both phthalates and the other substitutes), POLYSORB® isosorbide and POLYSORB® ID 46 did not induce any significant effect in the H295R steroidogenesis assay.

## 5. Conclusion

Except for DEHP which exhibited a moderate estrogenic activity in the E-Screen assay, in our results, none of the tested substances was estrogenic or antiestrogenic in the E-Screen assay, or androgenic or antiandrogenic in the AR transactivation assay in MDA-kb2 cells. Globally, both DEHP and DINP phthalates as well as DEHT, DINCH, and TOTM disrupted steroidogenesis in the H295R assay, mainly by inducing an increase in estradiol synthesis. In contrast, no such effect was observed for POLYSORB® isosorbide and POLYSORB® ID 46. Therefore, they represent interesting phthalate substitute candidates. When a comparison was possible, our results were globally similar to the available published data, meaning that this set of tests is quite robust and these *in vitro* data are relevant. The battery of *in vitro* assays was conducted in order to determine if there was, or not, any alert. In our opinion, in the case an alert was triggered, an *in vivo* confirmation would be needed before drawing a conclusion.

## Figures and Tables

**Figure 2:**
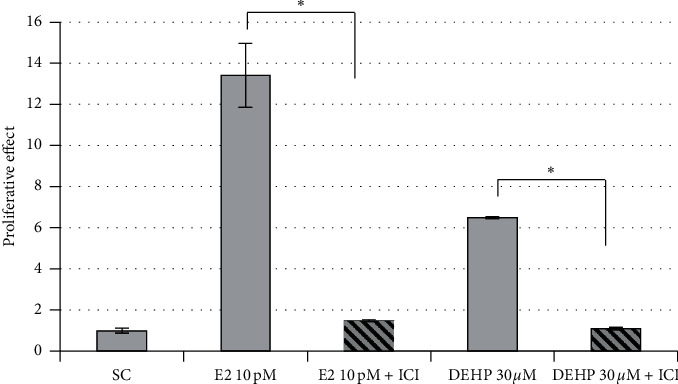
E-Screen agonist competitive confirmation assay for DEHP. MCF-7 cells were exposed for 120 h to DEHP 30 *μ*M with or without the reference antiestrogen ICI 182,780 (ICI). E2 10 pM was used as reference estrogenic control. *∗*Statistically significant decrease compared to the proliferation without ICI (*p* ≤ 0.05).

## Data Availability

The raw data used to support the findings of this study are available from the corresponding author upon request.
